# Effect of educational brochure compared with video on disease-related knowledge in patients with juvenile idiopathic arthritis: A randomized controlled trial

**DOI:** 10.3389/fped.2022.1048949

**Published:** 2022-12-09

**Authors:** Waraporn Sunthornsup, Soamarat Vilaiyuk, Sirisucha Soponkanaporn

**Affiliations:** ^1^ Department of Pediatrics, Faculty of Medicine Ramathibodi Hospital, Mahidol University, Bangkok, Thailand; ^2^ Division of Rheumatology, Department of Pediatrics, Faculty of Medicine Ramathibodi Hospital, Mahidol University, Bangkok, Thailand

**Keywords:** juvenile idiopathic arthritis, education, knowledge, randomized controlled trial, educational brochure, educational video

## Abstract

**Introduction:**

Patient education plays an important role in the management of chronic diseases such as juvenile idiopathic arthritis (JIA). This study compared the effectiveness of a brochure and a video regarding JIA-related knowledge immediately after the intervention, and at 4 weeks post-intervention.

**Methods:**

A prospective randomized controlled trial was conducted. Patients with JIA or parents were randomized to receive education from either a brochure (*n* = 50) or a video (*n* = 50) at the clinic. Participants answered questionnaires about disease-specific knowledge before the intervention (T0), immediately after the intervention (T1), and at follow-up 4 weeks later (T2). The questionnaire comprised 15 multiple-choice questions. Final scores ranged from 0 to 15, and were scaled from 0% to 100% to calculate the percentage of knowledge scores. Ninety participants completed the questionnaire at T2 (42 in the brochure and 48 in the video group).

**Results:**

The mean percentage of knowledge scores at T0 was not significantly different between the brochure group and the video group. At T1, the mean percentage of knowledge scores was significantly higher in the video group compared with the brochure group (86.7 ± 12.9% vs. 76.0 ± 21.4%, *p* = 0.003). Among parents with an educational level below secondary school, the mean percentage of knowledge scores at T1 was significantly higher in the video group compared with the brochure group (83.5 ± 14.4% vs. 69.1 ± 23.2%, *p* = 0.006). Participants in both groups had significantly higher mean percentage of knowledge scores at T2 compared with T0 (72.7 ± 20.3% vs. 51.1 ± 24.7%, *p* < 0.001 in the brochure group and 78.3 ± 15.7% vs. 56.1 ± 21.9%, *p* < 0.001 in the video group). There was no significant difference in the mean percentage of total score change between T2 and T1 between the brochure and video groups (−4.7 ± 13.3% vs. −8.5 ± 11.0%, *p* = 0.152).

**Conclusion:**

The video was more effective for improving disease-related knowledge immediately post-intervention, particularly in participants with limited education. Although both educational tools had lasting effects on knowledge, the retention rate declined at 4 weeks after both interventions.

**Trial registration:**

Thai Clinical Trials Registry (TCTR)20200310004, retrospectively registered since 06/03/2020

## Introduction

Juvenile idiopathic arthritis (JIA) is the most common rheumatic disease in children (1, 2). In Thailand, JIA accounts for 60% of pediatric patients with rheumatic diseases (3). The course of JIA is chronic and relapsing-remitting. Treatment of JIA is often complicated because of the chronicity and complexity of the disease, and the use of long-term medications. Therefore, patients also need continuous education during the course of the disease to ensure better disease management. JIA has a substantial impact on physical and functional disability, including chronic pain, joint deformities and growth impairments (4, 5). These factors can result in suboptimal quality of life and psychological well-being (6–9). JIA with disability can lead to negative school performance in childhood and unemployment in adulthood (10, 11).

One important aspect of managing JIA is enabling patients and family to participate in educational activities, because increased comprehension of the disease by patients and families is associated with better disease outcomes (12). In addition, educational programs can enhance health-related behavior and adherence to treatment, and minimize the total burden of disease (13–15). Specific knowledge can be delivered by physician-patient communication and educational tools such as brochures, comics, or videos (16–18). Because of the complexity of JIA, physician-patient communication might not be adequate for delivering sufficient information about the disease and medications. To improve patients' knowledge of JIA in Thailand, more accessible educational tools should be developed and implemented. Although comprehensive information about JIA in the Thai language is provided on the Paediatric Rheumatology International Trials Organisation (PRINTO) website (19), this detailed educational resource might be overwhelming for young patients and parents with a low level of education.

Although written materials, such as brochures, can improve patients' knowledge, these tools may not be useful for patients with low health literacy or socioeconomic disadvantage (20). Two studies reported that video materials were more effective than brochures for improving patient's knowledge (20, 21). However, no study or resource has been evaluated in pediatric patients with JIA. Therefore, we aimed to define the effectiveness of a brochure compared with a video regarding JIA-related knowledge immediately after the intervention and at 4 weeks post-intervention.

## Materials and methods

### Design and participants

**Figure 1:**
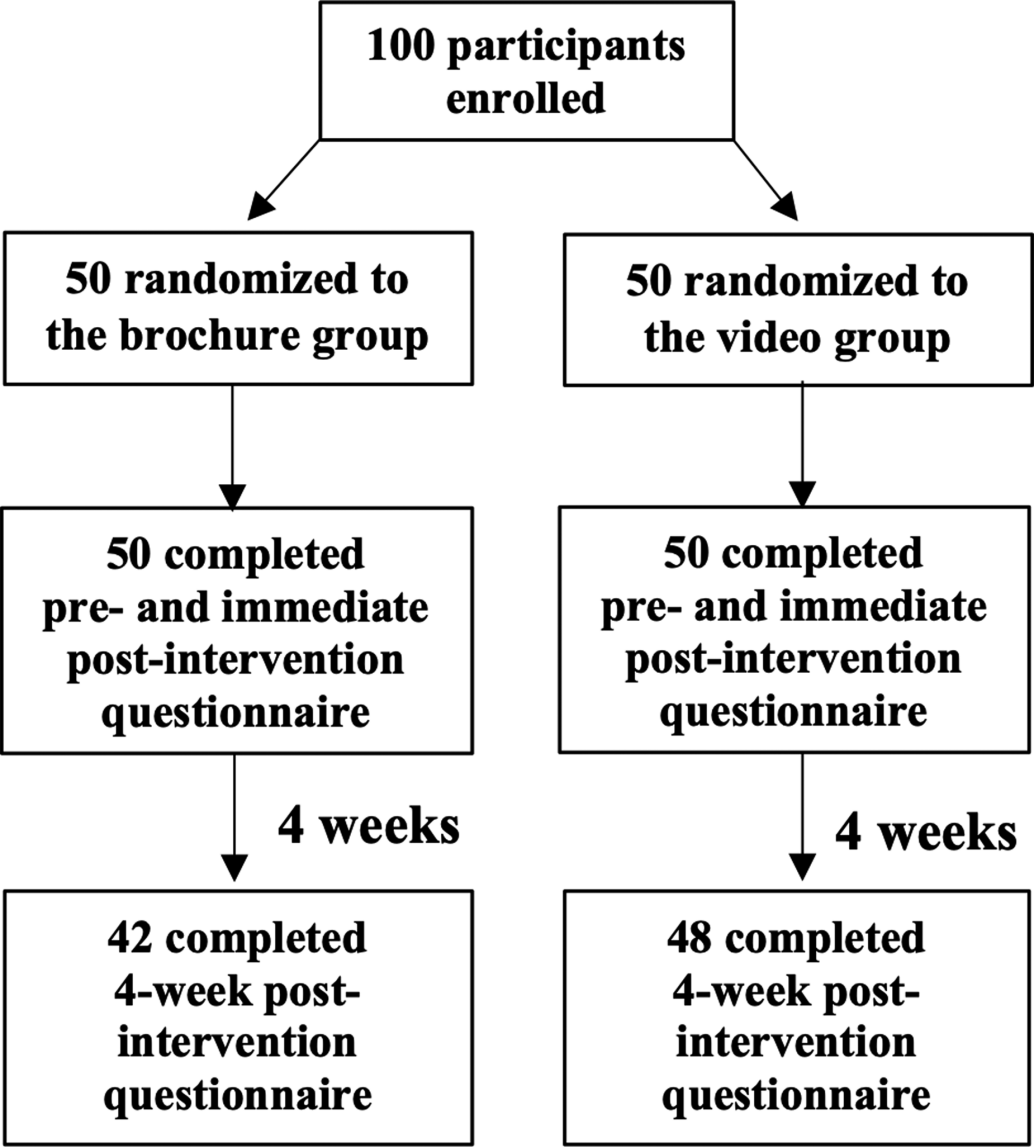
Flow chart of participant enrolment, allocation, and follow-up.

This randomized, controlled trial recruited JIA patients diagnosed by the International League of Associations for Rheumatology (ILAR) criteria (22) in the Pediatric Rheumatology Clinic at the Faculty of Medicine Ramathibodi Hospital. Data collection was conducted between December 2019 and December 2020. Inclusion criteria were either patients with JIA who had already completed at least the 8^th^ grade or the parents of patients who had not yet completed the 8^th^ grade, and all participants were able to read and communicate in Thai language. Patients living in family with medical personnel and patients who were deaf or blind (*n* = 1 each) were excluded. The questionnaire was answered either by patients who had already completed at least the 8^th^ grade, or by the parents of patients who had not yet completed the 8^th^ grade. Participants were randomized to either receive education from a brochure or from a video at the clinic (Figure 1). All participants answered a questionnaire regarding disease-specific knowledge before the intervention (pre-intervention, T0), immediately after the intervention (immediate post-intervention, T1), and at follow-up 4 weeks later (4-week post-intervention, T2). A questionnaire for assessing patients' satisfaction was also completed immediately at T1. Both groups received similar care and treatment in accord with standard guidelines (23–25). Informed consent was obtained from patients or their parents (if the patient was younger than 18 years).

Baseline demographic and clinical characteristics data, including age, gender, disease duration, patient's education level, parent's employment status, geographic region, JIA subtype, disease activity, and health-associated behaviors were collected from medical records and face-to-face interviews. Disease activity was assessed using the Juvenile Arthritis Disease Activity Score 27 (JADAS-27) (26) and Wallace criteria (27). JADAS-27 score was calculated by summing the scores of four criteria: physician's global assessment of disease activity, parents or patients' global assessment of well-being, the number of active joints, and erythrocyte sedimentation rate (ESR) calculated using the following formula: ESR (mm/h)−20/10. JADAS-27 scores ranged from 0 to 57, with higher scores reflecting more active disease (28). The Wallace criteria were used to define disease status in JIA patients, which was classified into active disease, inactive disease, clinical remission on medication, and clinical remission without medication. Criteria for inactive disease included: no active arthritis; no fever, rash, serositis, splenomegaly, or generalized lymphadenopathy attributable to JIA; no active uveitis; normal ESR or C-reactive protein (CRP) and; physician's global assessment indicates no disease activity. Clinical remission with medication was defined as patients who met the criteria for inactive disease for a minimum of six consecutive months while taking medication. Clinical remission without medication was defined as patients who met the criteria for inactive disease without taking any anti-inflammatory medications for a minimum of 12 consecutive months. Patients who did not meet inactive criteria were defined as active disease.

The study was approved by the Ethics Committee of the Faculty of Medicine Ramathibodi Hospital, Mahidol University, Thailand (ID MURA2019/904) and was conducted in accordance with the principles of the Declaration of Helsinki.

### JIA educational tools

The conceptual framework for developing the educational tools included reviewing data from previous studies focused on patient education in JIA (15, 16, 19, 29) and defining the key messages by WS, SV and SS. The included topic areas were modified to fit with the context of our patients. The preliminary version of educational brochure and video were developed. Both tools were reviewed by 10 laypeople and additional changes in design and wording were made. Then, cognitive interview was conducted with patients with JIA and their parents to refine the final version. Both tools had similar content and comprised four components including: 1) general knowledge and disease etiology; 2) treatment and adverse drug reactions; 3) self-care knowledge including physical activities, immunization and diet; and 4) disease relapse management (Supplementary Table S1). There were approximately 890 words in the information brochure, which took approximately 5–7 min to read. The duration of the animated video was 5 min. A pilot study was established in 10 participants and both educational tools showed good feasibility and acceptability. The final version of the brochure was in the Supplementary data S1 and the video was posted on the website of Department of Pediatrics, Faculty of Medicine Ramathibodi hospital, Mahidol university (https://www.rama.mahidol.ac.th/ped/th/vdo/special_disease/16sep2021-1054).

### Outcome measures

The primary outcome was the change in knowledge about JIA, which was evaluated using the JIA knowledge questionnaire. The knowledge questionnaire was developed following these steps including reviewing previous studies that demonstrated validated patient knowledge questionnaire for JIA. Data about questionnaire for JIA exist in the literature but they were not validated (15, 16). Thus, we had to extend our review on RA in adults, which had a validated patient knowledge questionnaire (29). The prototype questionnaire was derived from those previous studies. Key domains that were important for JIA patients were identified. We also followed the EULAR recommendations about the important content for patient education in JIA including the knowledge about theoretical disease and treatment (30). The questionnaire was adapted and modified to reflect custom and practice in Thailand. Cognitive interviews were performed with 5 JIA patients and 5 parents to identify any possibility of misunderstanding issues. The preliminary question was developed. Finally, validity and reliability of the questionnaire were tested. Face (performed in 10 participants) and curricular validity (performed in two pediatric rheumatologists, a general practitioner and a pediatric rheumatology nurse specialist) were applied to assess content validity. The contents of the questionnaire were subjectively matched with the objectives of our study. Pilot test was done in 20 participants (7 JIA patients and 13 parents of JIA patients). Test-retest reliability showed good consistency of measures with a single measure intraclass correlation coefficient of 0.985 with 95% confidence interval of 0.712–0.969 and Pearson's correlation coefficient of 0.985, *p *< 0.001. The Cronbach's alpha was 0.69 which demonstrated good internal consistency.

The questionnaire comprised 15 multiple-choice questions with a choice of five responses. The questionnaire included an “I don”t know” response option for each question to prevent guessing. The questionnaire included general knowledge (three items), treatment and adverse drug reactions (five items), self-care knowledge and immunization (four items) and disease relapse management (three items) (Supplementary data S2). For each question, the correct answer was given 1 point. The final score ranged from 0 to 15, which the percentage of knowledge scores was scaled from 0% to 100%. Lower percentages reflect poorer knowledge. Questionnaires for both patients or parents were similar with the same scoring system. The secondary outcome was knowledge retention 4 weeks after the intervention. We also investigated satisfaction regarding educational tools, which was evaluated using a 10 cm visual analog scale that can assess the overall satisfaction, usefulness, content clarity, content suitability, applicability, and attractiveness of content. The scores ranged from 0 (not satisfied) to 10 (most satisfied) and were scaled from 0% to 100%.

### Sample size and randomization

A sample size calculation was conducted on the basis of results from a previous study examining JIA-related knowledge (16). We expected that the percentage of knowledge scores after the intervention would improve by 33% in the video group and 20% in the brochure group. A sample size of 42 for each group would give 80% power to detect the differences using a two-sample *t*-test with 0.05 two-sided significance level. We recruited 100 participants in this study to provide acceptable statistical power. Block randomization (block size of four) was generated by a laboratory technician. Participants were randomly assigned to the brochure or video groups with a 1: 1 ratio. Allocation was concealed from participants.

### Statistical analysis

Continuous variables were shown as mean and standard deviation (SD) or median and interquartile range (IQR), as appropriate. Categorical variables were shown as number and percentages. Changes in mean knowledge scores between two time points were analyzed using a paired *t*-test or Wilcoxon's test, depending on the data distribution. Differences in the median change in scores from baseline between two independent groups were evaluated using a two-sample *t*-test or Mann-Whitney *U* test according to an intention-to-treat analysis. We considered a *P*-value less than 0.05 to indicate statistical significance. SPSS Statistics for Windows (Version 20.0, Armonk, NY, United States; IBM Corporation) was used to analyze the data.

## Results

A total of 100 patients with JIA answered the questionnaire before (T0) and immediately after the intervention (T1), with 50 participants in each intervention arm. Patients' mean (SD) age was 13.7 (4.9) years and 57% of participants were female. Systemic JIA was the most common subtype (37%), followed by polyarticular JIA (28%), and enthesitis-related arthritis (21%). Patients' demographic data and clinical characteristics are shown in Table 1. We found no significant differences between the two groups regarding JIA subtypes, disease duration, severity, or activity. At the 4-week post-intervention follow-up (T2), 42 participants in the brochure group and 48 participants in the video group completed the questionnaire (Figure 1).

**Table 1:** Baseline and clinical characteristics of 100 participants in the brochure and video groups.

Characteristics	Total (*N* = 100)	Brochure (*N* = 50)	Video (*N* = 50)	*P*
Female	57 (57%)	26 (52%)	31 (62%)	0.419
Age, years (mean, SD)	13.7 (4.9)	13.2 (4.6)	14.3 (5.1)	0.27
JIA subtypes				
Systemic JIA	37 (37%)	15 (30%)	22 (44%)	0.33
Polyarticular JIA	28 (28%)	14 (28%)	14 (28%)	
ERA	21 (21%)	12 (24%)	9 (18%)	
Oligoarticular JIA	12 (12%)	7 (14%)	5 (10%)	
Others	2 (2%)	2 (4%)	0 (0%)	
Mean disease duration, years (median, IQR)	5.1 (2.1, 9.3)	3.9 (2.1, 8.2)	6.5 (2.9, 9.6)	0.086
JADAS-27 (median, IQR)	3.0 (0, 7.9)	3.4 (0, 9.2)	1.9 (0, 7)	0.169
Wallace criteria				
Active disease	48 (48%)	26 (52%)	22 (44%)	0.473
Inactive disease	6 (6%)	3 (6%)	3 (6%)	
Clinical remission	46 (46%)	21 (42%)	25 (50%)	
Current medications				
DMARDs	42 (42%)	22 (44%)	20 (40%)	0.301
Biological DMARDs	26 (26%)	16 (32%)	10 (20%)	
Steroids only	4 (4%)	1 (2%)	3 (6%)	
NSAIDs only	3 (3%)	2 (4%)	1 (2%)	
None	25 (25%)	9 (18%)	16 (32%)	
Current patient education level				
Secondary school	56 (56%)	29 (58%)	27 (54%)	0.137
College	11 (11%)	3 (6%)	8 (16%)	
Grade point average*				
Below 3.0	32 (32%)	12 (24%)	20 (40%)	0.137
Above 3.0	35 (35%)	20 (40%)	15 (30%)	
Parent education level				
Below secondary school	58 (58%)	30 (60%)	28 (56%)	0.910
Vocational certificate	5 (5%)	2 (4%)	3 (6%)	
Above bachelor degree	37 (37%)	18 (36%)	19 (38%)	
Region				
Bangkok metropolitan	22 (22%)	7 (14%)	15 (30%)	0.053
Family income, THB/month				
Below 30,000	72 (72%)	34 (68%)	38 (76%)	0.330
Above 30,000	28 (28%)	16 (32%)	12 (24%)	
Parents’ occupation				
Self-employed	31 (31%)	17 (34%)	14 (28%)	0.150
Company’s employee	31 (31%)	10 (20%)	21 (42%)	
Housewife	13 (13%)	9 (18%)	4 (8%)	
Civil servant	15 (15%)	9 (18%)	6 (12%)	
Farmer	10 (10%)	5 (10%)	5 (10%)	
Answer questionnaire by				
Patients	67 (67%)	32 (64%)	35 (70%)	0.523
Parents	33 (33%)	18 (36%)	15 (30%)	

Data expressed as number (%), otherwise indicated.

* Only in self-answering patients.

DMARDs, disease-modifying anti-rheumatic drugs; ERA, enthesitis-related arthritis; IQR, interquartile range; JADAS-27, juvenile arthritis disease activity score 27; JIA, juvenile idiopathic arthritis; NSAIDs, non-steroidal anti-inflammatory drugs; THB, Thai baht (1 US dollar = 36 THB).

Regarding the level of education, 60% of parents of JIA patients had completed secondary school or below in the brochure group compared with 56% in the video group (*p *= 0.91). The average monthly family income per household was below 30,000 Thai baht for 68% of families in the brochure group and 76% of families in the video group (*p *= 0.33).

### Baseline knowledge scores

**Figure 2:**
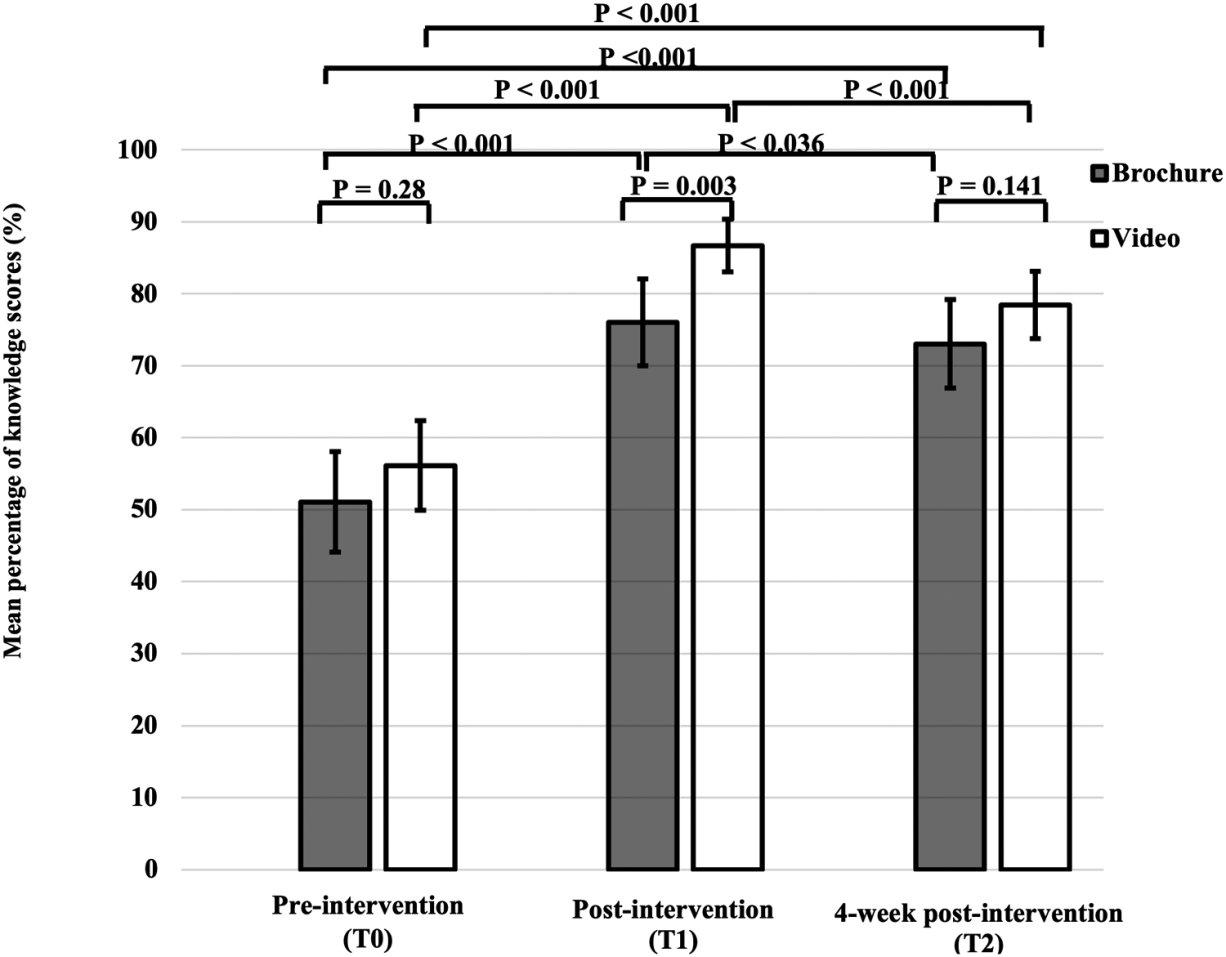
Total knowledge scores before (T0), immediately after intervention (T1) and at 4-week post-intervention (T2). Error bars are standard error.

The mean percentage of total knowledge scores at T0 were comparable between the brochure and the video groups (51.1 ± 24.6% vs. 56.1 ± 21.9%) (*p* = 0.28) (Figure 2). There were no significant differences in four domains of knowledge between the brochure and video groups (Table 2). The details of knowledge at T0 revealed that participants in both groups had limited knowledge regarding various topics, including immunization, disease relapse management, and steroid usage, with mean percentage scores of 20%, 29%, and 30%, respectively. Considering the effect of disease duration on disease knowledge, patients with a disease duration of more than 2 years still had limited knowledge at T0 (57.0 ± 22.4%). Additionally, patients in the video group tended to have longer disease duration than patients in the brochure group and patients in the video group tended to live in capital city than patients in the brochure group (Table 1). To adjust this possible confounder, multiple linear regression analysis was used and demonstrated that mean disease duration (standardized coefficient [*β*] 0.984, 95% confidence interval [CI] −0.506–2.474*, p* = 0.193) and region (*β* 1.120, 95% CI −0.838–3.078, *p *= 0.259) were not significantly associated with baseline knowledge scores (Supplementary Tables S2, S3).

**Table 2:** Mean knowledge scores in different domains before intervention (T0), immediately after intervention (T1), and at 4 weeks post-intervention (T2) between the brochure and video groups.

	T0			T1			T2		
	Brochure (mean, SD)	Video (mean, SD)	*P*	Brochure (mean, SD)	Video (mean, SD)	*P*	Brochure (mean, SD)	Video (mean, SD)	*P*
Total score (15)*	7.66 (3.70)	8.42 (3.29)	0.28	11.40 (3.21)	13.0 (1.94)	*0*.*003*	10.90 (3.04)	11.75 (2.35)	0.141
General knowledge (3)*	1.64 (0.99)	1.80 (0.97)	0.415	2.08 (0.85)	2.66 (0.52)	*<0*.*001*	2.26 (0.70)	2.40 (0.74)	0.381
Treatment and adverse drug reactions (5)*	2.46 (1.61)	2.70 (1.42)	0.43	3.82 (1.29)	3.98 (1.06)	0.499	3.43 (1.31)	3.73 (1.09)	0.237
Self-care knowledge and immunization (4)*	1.84 (1.17)	2.16 (1.13)	0.167	3.12 (1.04)	3.70 (0.58)	*0*.*001*	3.00 (0.96)	3.25 (0.76)	0.172
Disease relapse management (3)*	1.60 (0.83)	1.76 (0.94)	0.369	2.38 (0.86)	2.66 (0.63)	0.065	2.14 (0.81)	2.42 (0.71)	0.092

* Full scores.

SD, standard deviation.

### Improvement of disease-specific knowledge right after intervention

At T1, the mean percentage of total knowledge score in the video group was significantly higher than that in the brochure group (86.7 ± 12.9% vs. 76.0 ± 21.4%) (*p* = 0.003). Among the parents of JIA patients with educational level below secondary school, the mean percentage of total knowledge score at T1 was significantly higher in the video group than in the brochure group (83.5 ± 14.4% vs. 69.1 ± 23.2%) (*p* = 0.006). The mean percentage of total knowledge score at T1 showed a significant increase compared with that at T0 in both intervention groups (*p* < 0.001) (Figure 2).

The mean total score difference between T1 and T0 in the video group tended to be higher than that in the brochure group, but this difference was not significant (4.44 ± 2.68 vs. 3.74 ± 3.26, *p* = 0.245) (Table 3). Participants in the video group showed a greater improvement in some knowledge domains, including general knowledge, self-care and immunization, and disease relapse management, compared with the brochure group. However, compared with the brochure group, only the general knowledge domain showed a significantly greater improvement of knowledge in the video group (28.7% vs. 14.7%, *p* = 0.018).

**Table 3:** Mean score differences between immediately post-intervention and before the intervention (T1–T0), 4 weeks post-intervention and immediately post-intervention (T2–T1), and 4 weeks post-intervention and before the intervention (T2–T0).

	Mean T1–T0 (SD)		*P*	Mean T2–T1 (SD)		*P*	Mean T2–T0 (SD)		*P*
	Brochure	Video		Brochure	Video		Brochure	Video	
Total score	3.74 (3.26)	4.44 (2.68)	0.245	−0.71 (2.00)	−1.27 (1.65)	0.152	3.14 (2.77)	3.23 (2.94)	0.887
General knowledge	0.44 (0.88)	0.86 (0.86)	*0*.*018*	0.17 (0.62)	−0.27 (0.79)	*0*.*005*	0.55 (0.86)	0.56 (0.94)	0.938
Treatment and adverse drug reactions	1.36 (1.60)	1.28 (1.21)	0.779	−0.52 (1.04)	−0.23 (0.93)	0.159	0.98 (1.35)	1.02 (1.35)	0.876
Self-care knowledge and immunization	1.28 (1.16)	1.54 (1.20)	0.273	−0.12 (1.02)	−0.48 (0.77)	0.06	1.12 (1.09)	1.06 (1.16)	0.812
Disease relapse management	0.78 (0.95)	0.90 (1.02)	0.544	−0.31 (0.84)	−0.23 (0.69)	0.62	0.52 (0.92)	0.65 (1.19)	0.592

SD, standard deviation.

### Knowledge retention at 4 weeks post-intervention

Participants in both groups had higher knowledge scores at T2 compared with T0 in all aspects of knowledge (Table 2). However, the mean percentage of total knowledge scores at T2 was significantly lower than that at T1 in both intervention groups (72.7 ± 20.3% vs. 76 ± 21.4%, *p* = 0.036 in the brochure group and 78.3 ± 15.7% vs. 86.7 ± 12.9%, *p* < 0.001 in the video group) (Figure 2). There was no significant difference in the mean total score change between T2 and T1 in the brochure and the video groups (−0.71 ± 2.00 vs. −1.27 ± 1.65, *p* = 0.152) (Table 3). Interestingly, participants in the brochure group retained more general knowledge compared with the video group (0.17 ± 0.62 vs. −0.27 ± 0.79, *p* = 0.005). However, there was no significant difference in knowledge retention at 4 weeks in other areas of knowledge between groups. Regarding the details of retention in the general knowledge questions, participants in the brochure group had better knowledge of JIA symptoms with mean percentage scores of 35%, 60%, and 78% at T0, T1, and T2, respectively. In contrast, participants in both groups had less knowledge retention regarding treatment and adverse drug reactions at 4 weeks, particularly regarding steroid (mean percentage scores: 44% at T2 vs. 55% at T1) and methotrexate usage (mean percentage scores: 57% at T2 vs. 64% at T1).

### Satisfaction with the educational tools

Participants in both groups were highly satisfied with the educational tools, with overall satisfaction scores of 92.4% in the brochure group and 94.1% in the video group (*p *= 0.53) (Table 4). Regarding each domain in the satisfaction questionnaire, satisfaction in the video group was between 89% and 93% in all domains, which tended to be higher than that in the brochure group (84%–90%) with *P*-values between 0.20 and 0.35.

**Table 4:** Patient satisfaction between the brochure and the video groups.

Satisfaction domain	Satisfaction scores (%)		*P*
	Brochure (*N* = 50)	Video (*N* = 50)	
Overall satisfaction	92.4	94.1	0.530
Usefulness	88.0	91.3	0.290
Content clarity	84.3	89.1	0.200
Content suitability	88.5	92.7	0.210
Applicability	88.6	92.3	0.240
Attractiveness of content	89.9	93.1	0.350

## Discussion

This study aimed to compare the effects of a newly developed brochure and video in communicating disease-related knowledge to JIA patients. The current findings indicated that disease-related knowledge among JIA patients and their caregivers could be improved by reading an educational brochure or watching a video. However, there was a greater increase in general knowledge scores immediately after the intervention in the video group compared with that in the brochure group. The improvement of knowledge was even more pronounced among participants with a lower level of education, particularly in the video group. These findings suggest that participants with limited education might benefit more from educational tools that are entertaining, simple, and involve a combination of text and animation, such as a video. Previous studies have reported that the combination of multimedia images, voice, and text had more positive effects on patient's disease-related knowledge and awareness of disease compared with a text-only educational tool (20, 21).

Our findings are in accordance with those of Beaujean and colleagues, which demonstrated the effects of a leaflet and a movie on knowledge about Lyme disease (31). In that study, the results revealed that participants in both the leaflet and the movie group had greater knowledge than those in the control group, and knowledge scores were highest in the movie group. However, knowledge about Lyme disease was not retained after 1-month post-intervention in both the leaflet and movie groups. The current findings indicated that there was an immediate improvement of knowledge after both interventions. Although there was a lasting effect on disease-related knowledge at 4 weeks post-intervention, the 4-week retention rate decreased significantly from the immediate post-intervention test in both groups. This finding is in accord with a study by Ebbinghaus, which demonstrated a forgetting curve pattern in the decline of retention rate over several months after meaningful information was presented (32). In contrast, Mendelson and colleagues reported that JIA-related knowledge increased significantly after reading a comic book, and this knowledge was retained after 1 month and also at 1 year (16). The difference between these previous results and the current findings could potentially be explained by the lower level of education of caregivers and lower socioeconomic status of families in the current study compared with the previous study. However, the dropout rate in Mendelson et al.'s study at 1-year follow-up was above 50% (16). Thus, participants who showed up at 1-year follow-up might have been more concerned about their health and disease status compared with those who did not. Another possible reason for the lower knowledge retention in the current study was that patients and parents were not able to choose the educational tools that they preferred because they were randomly allocated to an intervention group. Schulz et al. reported that the effectiveness of educational tools depends on users' personal preferences (33). In addition, in our protocol, participants were able to access the brochure and the video only once at the clinic. Repeated use of the brochure or the video may have led to longer-lasting effects on knowledge retention. Moreover, providing an educational video program in the clinic while patients were waiting for their appointment could improve patients' disease-related knowledge and promote positive outpatient visit experiences.

The current study also showed that JIA patients with a disease duration of longer than 2 years still possessed limited knowledge about JIA at baseline. This finding could potentially be explained by the limited number of pediatric rheumatologists in Thailand, which may mean that rheumatologists spend little time with patients with longer disease duration and stable disease activity. This could potentially lead to the delivery of inadequate information about disease and medications to patients and parents. Additionally, approximately 70% of participants in our study had monthly household income lower than the Thai average monthly income, and approximately 60% of parents of JIA patients had no education beyond secondary school. Previous studies have reported that lower health literacy can result in disadvantageous self-management skills and inappropriate health-related decisions (33, 34). In this context, an alternative educational tool that is simple and accessible should be developed to enhance disease-related knowledge. Knowledge regarding immunization at baseline was very limited, possibly because the mean age of our patients was 13.8 years, meaning that they had less recent experience of receiving vaccines compared with younger children. Another possible reason is that physicians may not have been concerned about providing advice associated with immunization, which should be undertaken with all older children and adolescents. To address this issue, physicians should provide more information about vaccination, particularly regarding vaccines for influenza and invasive pneumococcal disease in all patients.

For the development of educational tools, few models are available for developing reproducible and rigorous designs. In recent years, behavioral intervention development models have been described (35, 36). One example, The Obesity Related Behavioral Intervention Trials (ORBIT) model, was applied retrospectively to the process of developing and evaluating our educational tools (35). Key steps of intervention development include translating clinical theory to an initial design for educational content (phase 0), defining and refining the content (phase 1), establishing feasibility and acceptability (phase 2), efficacy trials (phase 3), and establishing effectiveness (phase 4). Applying this conceptual framework, our educational tool development process has addressed phases 0 to 2. However, we have not yet conducted multicenter effectiveness trials in patients with JIA (i.e., phase 3). Therefore, our educational tools may have limited generalizability until further multicenter studies are conducted.

This study emphasizes the need to strengthen educational programs in chronic disease management. It may be useful for future studies to evaluate disease activity and health-related behaviors (e.g., medication compliance and treatment adherence) after receiving educational tools that could help to alleviate disease outcome. Moreover, systemic JIA which was the most common subtype in this study, was phenotypically different from other subtypes. Patients with active disease of systemic JIA might have fever and salmon rash rather than arthritis. Thus, our educational materials might be inadequate to apply in all JIA subtypes. In the future, the educational materials should be specifically designed for systemic JIA. Additionally, the educational materials might be more valuable and applicable if they could be reviewed by pediatric rheumatologists from other medical centers.

This study involved several limitations. First, because we included both newly diagnosed patients with JIA and follow-up patients who had been diagnosed for some time, the disease duration may have affected baseline disease knowledge. However, we made an effort to randomize participants and found no significant effect of disease duration between the brochure and video groups. Moreover, we cannot exclude the possibility that participants answered the questionnaire using knowledge obtained from other educational resources during the study period. We believe that this factor did not adversely affect the representativeness of 4-week post-intervention knowledge in this study.

In conclusion, both the brochure and the video increased JIA-related knowledge and participants were satisfied with both resources. The video was more effective for improving disease-related knowledge immediately post-intervention. Although both educational tools had lasting effects on knowledge, the retention rate declined 4 weeks after both interventions. Repeated exposure to the educational tools in a regular educational program may increase their effectiveness.

## Data Availability

The raw data supporting the conclusions of this article will be made available by the authors, without undue reservation.
