# Supplementary material for: Effect of educational brochure compared with video on disease-related knowledge in patients with juvenile idiopathic arthritis: A randomized controlled trial
Source: Front Pediatr. 2022 Dec 9;10:1048949. doi: 10.3389/fped.2022.1048949 (PMC9780585; doi:10.3389/fped.2022.1048949)
Supplement: Supplementary file 1 [file Datasheet1.pdf]

อะไรคือสาเหตุของการเกิดโรค JIA?

เกิดจากความผิดปกติของระบบภูมิคุ้มกันที่ทำงานมากผิดปกติจึงไปทำลายข้อและเกิดการอักเสบของข้อตามมา

เกิดอะไรขึ้นบ้างเมื่อเป็นโรค JIA?

จะทำให้เกิดการสร้างน้ำในข้อเพิ่มขึ้นและเนื้อเยื่อรอบๆข้ออักเสบ ทำให้ปวดข้อ ข้อบวมและทำให้ข้อติดตามมา หากข้อติดเป็นระยะเวลานาน ข้อจะผิดรูปและกล้ามเนื้อลีบ

อาการของโรค JIA มีอาการอย่างไร?

- **ปวด บวม ของข้อได้ทุกข้อ**
  มีข้อติดช่วงเช้าได้ หลังจากขยับข้อซัก 20-30 นาที อาการข้อติดจะค่อยๆดีขึ้น
- **เดินกะเผลก**
  หรือในเด็กเล็กๆจะไม่ยอมเดินจะขอให้อุ้มตลอดเวลา
- **เจ็บส้นเท้า หรือฝ่าเท้า**
- **อ่อนเพลีย เหนื่อยง่าย**
- **เบื่ออาหาร น้ำหนักลด**
- **อาจจะมีไข้สูงและมีผื่นบริเวณผิวหนัง**

* ในกรณีที่ปล่อยให้มีข้ออักเสบเรื้อรังเป็นเวลานาน ข้อจะโดนทำลายและทำให้กระดูกเจริญผิดปกติได้

รู้ได้อย่างไรว่าอาการโรค JIA กำเริบ?

ผู้ป่วยอาจจะมีอาการไข้ ปวดข้อ อ่อนเพลีย ไม่สุขสบาย ซึ่งอาจจะเป็นอาการของโรค JIA ที่กำเริบ

อะไรทำให้โรค JIA กำเริบได้?

- การพักผ่อนไม่เพียงพอ
- ความเครียด
- การติดเชื้อ
- รวมทั้งการกินยาไม่สม่ำเสมอ

จะทำอย่างไรเมื่อโรค JIA กำเริบ?

การปฐมพยาบาลเบื้องต้น ทำได้โดยประคบร้อนหรือเย็นบริเวณข้อที่ปวด กินยาลดไข้/ยาแก้ปวด แต่อย่างไรก็ตามหากดูแลเบื้องต้นไม่ดีขึ้น ควรไปพบแพทย์ทันที ห้ามปรับเพิ่มหรือลดยาเองเป็นอันขาด

Juvenile Idiopathic Arthritis หรือ JIA

ข้อมูลจาก : หน่วยโรคข้อและรูมาติสซั่ม
ภาควิชากุมารเวชศาสตร์ คณะแพทยศาสตร์
โรงพยาบาลรามาธิบดี มหาวิทยาลัยมหิดล

โรค Juvenile Idiopathic Arthritis หรือ JIA คืออะไร?

โรค JIA เป็นโรคที่มีข้ออักเสบเรื้อรังในเด็กที่อายุต่ำกว่า 16 ปี โดยมีอาการมากกว่า 6 สัปดาห์ ผู้ป่วยจะมีอาการปวด บวมของข้อและข้อติด

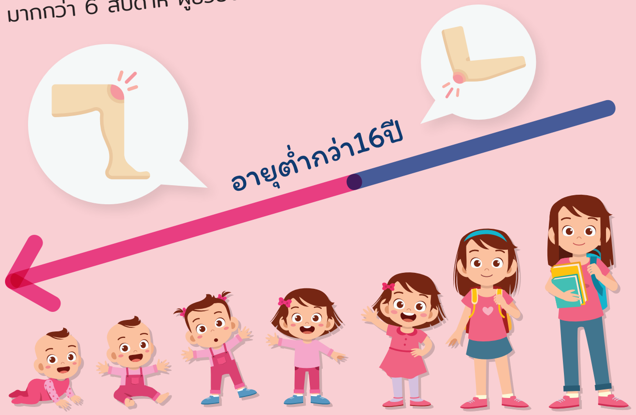

ทำไมต้องรักษาโรค JIA?

1. เพื่อลดอาการปวดข้อ
2. ป้องกันข้อติดและข้อโดนทำลาย

อาการทางตา
ที่สามารถพบได้ในโรค JIA คืออะไร?

ม่านตาอักเสบ ทำให้ตามองไม่ชัด
อาจมีตาแดงหรือไม่แดงก็ได้ ไม่มีขี้ตา

ยาที่ใช้ในการรักษามีอะไรบ้าง

1 ยาต้านการอักเสบ

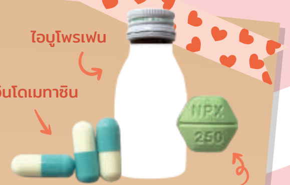

เช่น นาพรอกเซน (naproxen), อินโดเมทาซิน (indomethacin) และ ไอบูโพรเฟน (ibuprofen) ยานี้ช่วยลดการอักเสบและลดอาการปวด บวม แต่อาจเกิดอาการข้างเคียง เช่น ปวดท้อง จึงต้องกินหลังอาหารทันที และเนื่องจากยาขับออกทางไตจึงต้องดื่มน้ำเยอะๆ หากมีภาวะขาดน้ำ เช่น ท้องเสีย อาเจียน ให้หยุดยา

2 การฉีดยาเข้าข้อ

ยาที่ใช้ฉีดคือ ยาในกลุ่มสเตียรอยด์ (steroid) ซึ่งจะช่วยลดการอักเสบได้เร็ว ยาจะออกฤทธิ์ได้นานประมาณ 1-3 เดือน การฉีดยาเข้าข้อเป็นการรักษาที่ใช้เสริมร่วมกับการรักษาอื่นๆ หลังฉีดยาเข้าข้อให้พักการใช้ข้อนั้นๆอย่างน้อย 24 ชม.

3 ยาสเตียรอยด์ (Steroids)

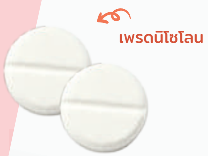

เป็นยากดภูมิคุ้มกันที่ใช้ได้ผลดีในผู้ป่วย JIA โดยเฉพาะผู้ป่วยที่มีปวดข้อร่วมกับไข้ ผลข้างเคียงที่พบบ่อย คือ อยากอาหารบ่อย ทำให้อ้วน กระดูกบาง ติดเชื้อง่าย การหยุดยาเองอาจอันตรายถึงแก่ชีวิต ดังนั้นควรใช้ยาอย่างสม่ำเสมอ

4 ยากดภูมิคุ้มกัน (DMARDs)

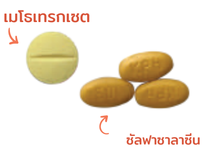

ยาชนิดนี้จะช่วยลดการอักเสบของข้อและควบคุมโรค ยาจะออกฤทธิ์ช้า จำเป็นต้องใช้อย่างสม่ำเสมอ ยากลุ่มนี้ ได้แก่

- **เมโธเทรกเซต (methotrexate)** ผลข้างเคียงที่พบบ่อย คือ คลื่นไส้ อาเจียน ปวดมวนท้อง และมีผลต่อตับ ทำให้ตับอักเสบได้ จึงต้องมีการติดตามผลเลือดดูการทำงานของตับเป็นระยะ การให้วิตามินโฟลิก ร่วมด้วย จะช่วยลดอาการตับอักเสบจากยาเมโธเทรกเซตได้ ดังนั้นต้องกินวิตามินโฟลิก อย่างสม่ำเสมอ
- **ซัลฟาซาลาซีน (sulfasalazine)** ผลข้างเคียงที่พบบ่อย คือ คลื่นไส้ อาเจียน และมีผลต่อตับ แต่มักไม่รุนแรงเท่ากับยาเมโธเทรกเซต

การดูแลตัวเอง
เมื่อเป็นโรค JIA ทำได้อย่างไรบ้าง?

- **การออกกำลังกาย**
  มีความสำคัญในการทำให้ข้อ กล้ามเนื้อและกระดูกแข็งแรง แนะนำการออกกำลังกายที่ไม่ลงน้ำหนักมากนัก ได้แก่ การว่ายน้ำ การปั่นจักรยาน
- **นอนหลับพักผ่อนให้เพียงพอ** อย่างน้อย 6-8ชั่วโมงต่อวัน
- **กินอาหารให้ครบ 5 หมู่**
  กินอาหารที่มีแคลเซียม เช่น นมจืด ผักใบเขียว ให้เพียงพอ
- **ให้ใส่หน้ากากอนามัยเมื่อเข้าสู่ที่ชุมชน**
  ไม่ใกล้ชิดผู้ป่วยโรคทางเดินหายใจหรือโรคติดต่อต่างๆและล้างมือบ่อยๆ สามารถไปโรงเรียนได้ตามปกติ
- **ห้ามฉีดวัคซีนชนิดเชื้อเป็นขณะที่กินยากดภูมิคุ้มกันเด็ดขาด**
  (ได้แก่หัด หัดเยอรมัน คางทูม) เนื่องจากจะทำให้เกิดโรคจากวัคซีน แต่ควรฉีดวัคซีนป้องกันไข้หวัดใหญ่เป็นประจำทุกปีและสำหรับวัคซีนชนิดอื่น ควรอยู่ภายใต้คำแนะนำของแพทย์
- **กินยาสม่ำเสมอตามแพทย์สั่งและต้องมาพบแพทย์ตามนัด**
  ห้ามปรับเพิ่มหรือลดยาเองเพราะอาจทำให้อันตรายถึงแก่ชีวิต
