# Supplementary material for: Effect of educational brochure compared with video on disease-related knowledge in patients with juvenile idiopathic arthritis: A randomized controlled trial
Source: Front Pediatr. 2022 Dec 9;10:1048949. doi: 10.3389/fped.2022.1048949 (PMC9780585; doi:10.3389/fped.2022.1048949)
Supplement: Supplementary file 2 [file Datasheet2.pdf]

Supplementary Data 2

The knowledge questionnaire consisted of four domains

<u>General knowledge domain</u>

1. What is juvenile idiopathic arthritis (JIA)?

A. A disease involving joint inflammation caused by inadequate nutrients.

B. A disease in which the immune system malfunctions and attacks the joints.

C. A hereditary disease in which the patient's relatives also have arthritis.

D. A disease caused by heavy exercise.

E. I don't know.

2. What are the causes of JIA?

A. An abnormal immune system attacking the joints and joint compositions.

B. Bacteria and/or viruses infecting the body and destroying joints and joint compositions.

C. Overuse of the joints resulting in inflammation of the joints.

D. A bacteria called JIA destroying white blood cells.

E. I don't know.

3. What are the symptoms of patients with JIA?

A. Pain, stiffness, and swelling in the joints.

B. Joint deformities, if the disease is left untreated.

C. Persistent fever.

D. A, B and C.

E. I don't know.

Treatment and adverse drug reactions domain

4. What is the best reason to treat JIA?

A. To reduce joint pain and stiffness, prevent joint damage and deformities, and improve the patient's quality of life.

B. To control the symptoms and perform joint surgery in the future.

C. To prevent the patient from spreading JIA to other people.

D. To restore normal immune function.

E. I don't know.

5. Which of the following are medications for treating JIA?

A. Non-steroidal anti-inflammatory drugs (NSAIDs), disease-modifying drugs (DMARDs), steroids, and biologics.

B. Antipyretic drugs.

C. Antibacterial drugs.

D. Antiviral drugs.

E. I don't know.

6. What are the indications and benefits of intraarticular steroid injection?

A. Used in patients with few inflamed joints. Rapid onset and long duration (1–3 months).

B. Used only in knee joints. Rapid onset and easy to use.

C. Used in patients with multiple inflamed joints. Can repeatedly inject every month.

D. Can be used in patients with a high-grade fever.

E. I don't know.

7. Which statement is not true about the use of steroids in JIA?

A. Steroids increase appetite, so the patients must limit high-energy food intake.

B. Prolonged use or high dosage of steroids can result in short stature and osteoporosis.

C. Steroids can suppress the immune system, and patients can have an increased risk of infection.

D. Patients can stop taking steroids by themselves.

E. I don’t know.

8. Which statement is not true about the use of methotrexate in JIA?

A. If you have any unusual symptoms, such as nausea, vomiting, or rash, stop taking the drug and go to the hospital.

B. Taking folic acid can reduce the side effects of methotrexate.

C. Methotrexate can cause abnormal liver enzymes, so blood tests are required.

D. Patients can decrease or increase the dose by themselves when symptoms improve or worsen.

E. I don’t know.

Self-care knowledge and immunization domain

9. Which statement regarding physical exercise in patients with JIA is true?

A. Patients who have no joint pain can exercise by starting with sports that are less impactful on the joints, such as swimming or cycling.

B. Patients who have no joint pain can do indoor exercise, such as boxing, football and Taekwondo.

C. Patients can’t exercise because they might have an accident while playing sports and cause permanent joint disability.

D. JIA patients can’t exercise because it may affect joints and aggravate the disease.

E. I don’t know.

10. Which statement regarding diet in JIA patients is true?

A. Patients are not allowed to eat poultry and some vegetables such as cha-om and Siamese neem tree.

B. Patients should eat meat, eggs, fish, and calcium-rich foods such as milk, vegetables, and fruits.

C. Patients should drink more than 2 L of milk per day.

D. Patients should eat a low-salt diet to prevent limb edema.

E. I don’t know.

11. Which statement is true about vaccination in JIA patients?

A. Patients cannot receive live vaccines while taking immunosuppressive drugs.

B. Patients can receive all vaccines, but they need to be observed in the hospital after vaccine injection.

C. Patients cannot receive any vaccines because they may aggravate JIA disease.

D. Patients can receive all vaccines because they can prevent infection.

E. I don’t know.

12. What should JIA patients do when going to a public place?

A. Patients should always wear a surgical mask, avoid close contact with people with respiratory illnesses, and wash their hands often.

B. While patients are receiving immunosuppressants, they should not go to school.

C. Patients should avoid going out.

D. Patients can live a normal life.

E. I don’t know.

Disease relapse management domain

13. What are the symptoms of JIA disease flare-up?

A. Pain, swelling, redness, warmth in various joints, and fever.

B. Red, itchy rash on various parts of the body.

C. Headache and dizziness.

D. High-grade fever, and possible seizure.

E. I don’t know.

14. What causes JIA disease to flare up?

A. Infection and stress.

B. Poor medication compliance.

C. Inadequate sleep.

D. A, B and C.

E. I don’t know.

15. What is the most appropriate management approach when JIA disease flares up (for example, joint pain)?

A. Patient should increase steroids and/or DMARDs by themselves and go to the hospital if symptoms do not improve.

B. Apply a cold or warm compression to sore joints. Take pain relief drugs and go to the hospital if symptoms do not improve.

C. Observe symptoms for 24 hours and go to the hospital if symptoms do not improve.

D. Rest and eat a healthy diet.

E. I don’t know.
