# Supplementary material for: Effect of educational brochure compared with video on disease-related knowledge in patients with juvenile idiopathic arthritis: A randomized controlled trial
Source: Front Pediatr. 2022 Dec 9;10:1048949. doi: 10.3389/fped.2022.1048949 (PMC9780585; doi:10.3389/fped.2022.1048949)
Supplement: Supplementary file 3 [file Table1.pdf]

Supplementary Table S1. The details of contents in the educational brochure and the video

Content	Brochure	Video
The description of JIA and its causes	Page 1 and 2	At 0.07-0.50
Signs and symptoms of JIA	Page 2	At 1.10-1.23
The importance and the goals of treatment	Page 3	At 1.26-1.38
Complications of JIA: joint deformity, uveitis	Page 3	At 0.54-1.09
Drugs and their adverse effects: NSAIDs, steroids including intraarticular steroid injection, DMARDs (methotrexate, sulfasalazine)	Page 4 and 5	At 1.39-3.20
Everyday life activity: exercise, diet, sleep	Page 5	At 3.22-3.49
Vaccinations in JIA patients	Page 5	At 3.50-4.00
Triggers of active disease: infection, stress, inadequate sleep, etc.	Page 6	At 4.10-4.25
How to do when patients have an active disease	Page 6	At 4.27-4.56
