# Supplementary material for: Effect of educational brochure compared with video on disease-related knowledge in patients with juvenile idiopathic arthritis: A randomized controlled trial
Source: Front Pediatr. 2022 Dec 9;10:1048949. doi: 10.3389/fped.2022.1048949 (PMC9780585; doi:10.3389/fped.2022.1048949)
Supplement: Supplementary file 4 [file Table2.pdf]

Supplementary Table S2. Possible confounders associated with knowledge score at T0

Variable	Standardized coefficient (β)	95%CI	*P*
Patient / parent respondent	-0.398	-10.3 – 9.504	0.937
Disease duration	0.984	-0.506 – 2.474	0.193
Age	0.151	-1.988 – 2.290	0.889
JIA subtype	0.067	-3.077 – 3.211	0.966
Region	1.120	-0.838 – 3.078	0.259
