# Supplementary material for: Effect of educational brochure compared with video on disease-related knowledge in patients with juvenile idiopathic arthritis: A randomized controlled trial
Source: Front Pediatr. 2022 Dec 9;10:1048949. doi: 10.3389/fped.2022.1048949 (PMC9780585; doi:10.3389/fped.2022.1048949)
Supplement: Supplementary file 5 [file Table3.pdf]

Supplementary Table S3. Possible confounders associated with knowledge score differences between T1-T0

Variable	Standardized coefficient (β)	95%CI	*P*
Patient / parent respondent	-0.049	-2.083 – 1.464	0.73
Disease duration	-0.060	-0.255 – 0.160	0.65
Age	-0.045	-0.219 – 0.163	0.773
JIA subtype	-0.064	-0.574 – 0.320	0.573
Region	-0.007	-0.271 – 0.254	0.949
